# Socially assistive robots and meaningful work: the case of aged care

**DOI:** 10.1057/s41599-025-05498-0

**Published:** 2025-07-11

**Authors:** Cristina Voinea, Tenzin Wangmo

**Affiliations:** 1 University of Oxford, Oxford, UK; 2 University of Basel, Basel, Switzerland

**Keywords:** Health humanities, Philosophy, Ethics

## Abstract

As socially assistive robots (SARs) become increasingly integrated into aged care, it becomes essential to ask: how do these technologies affect caregiving work? Do SARs foster or diminish the conditions conducive to meaningful work? And why does it matter if SARs make caregiving more or less meaningful? This paper addresses these questions by examining the relationship between SARs and the meaningfulness of care work. It argues that SARs should be designed to foster meaningful care work. This presupposes, as we will argue, empowering caregivers to enhance their skills and moral virtues, helping them preserve a sense of purpose, and supporting the integration of caregiving with other aspects of caregivers’ personal lives. If caregivers see their work as meaningful, this positively affects not only their well-being but also the well-being of care recipients. We begin by outlining the conditions under which work becomes meaningful, and then we apply this framework to caregiving. We next evaluate how SARs influence these conditions, identifying both opportunities and risks. The discussion concludes with design recommendations to ensure SARs foster meaningful caregiving practices.

## Introduction

By 2050, the global population aged 65 and above will double relative to its 2021 figures (UN Social Report 2023). As life expectancy increases, so does the prevalence of age-related health conditions such as dementia, frailty, and chronic illness (Jaul and Barron 2017; Fulop et al. 2010). Because of this demographic change, there will be a growing demand for caregiving services in the immediate future, as well as in the long term. Even today, and increasingly in the future, the supply of caregiving services falls short of rising demand (Addati 2018a). In this context, we need to find novel, innovative approaches to caregiving, not just in formal care environments such as nursing homes, but also in domestic care settings.

So, how can we adequately support the increasing number of older adults who require ongoing care? The integration of robots into caregiving practices offers prospects for addressing the challenge of a paucity of human caregivers amidst a surging demand.

Robots designed to care for children, older adults, people with disabilities, or individuals with medical conditions are called socially assistive robots (SARs). These robots are designed to be autonomous and to have natural interactions with patients, clinical staff, caregivers, or more generally, care recipients (Cifuentes et al. 2020). SARs can generally be (1) service robots, which assist caregivers in physical care, such as monitoring, lifting people or assisting them with mobility, hygiene or feeding; or (2) companion robots, which communicate and engage with care recipients to alleviate loneliness, prevent cognitive decline or to provide companionship (Abdi et al. 2018).

The ethical considerations surrounding the use of SARs in older adults’ care received considerable attention, touching upon issues of privacy, autonomy, the risk of harm, and the nature of care received (Poulsen et al. 2018; Vallor 2017; Elder 2016; Sharkey and Sharkey 2012; Sparrow and Sparrow 2006a). There are also fears expressed regarding the competence of robots in meeting the complex, individualized demands of older adults, as increasing reliance on technology could lead human caregivers to neglect the human bond and compassion they should provide care recipients with (Sparrow and Sparrow 2006b; Sharkey and Sharkey 2012). Researchers also investigated how these technologies are perceived by and affect older adults (Søraa et al. 2023; Jecker 2021; Pu et al. 2019; Vandemeulebroucke et al. 2018). But far less attention has been paid to how SARs affect caregivers themselves. And in those instances where caregivers are acknowledged, the concern is usually centered on ensuring SARs can relieve the “burden of care” (Sharkey and Sharkey 2012; Borenstein and Pearson 2010; Sharkey and Sharkey 2010).

However, caregiving is not merely a stress and a burden; it can also be a meaningful form of work that fosters skills, virtues, purpose, and can be integrated into one’s identity (Vallor 2017). When care work is considered meaningful, this contributes to caregivers’ well-being, enhances job satisfaction, and ultimately improves the quality of care for older adults and implicitly their well-being (Butcher and Buckwalter 2002; Ayres 2000; Noonan and Tennstedt 1997). As SARs increasingly become collaborators in aged care, it is essential to ask: how do these technologies affect caregiving work? Do SARs enhance or diminish the meaningfulness of caregiving?

Our paper addresses these questions by examining the relationship between SARs and the meaningfulness of care work. It argues that SARs should be designed primarily to foster meaningful care work by empowering caregivers to better their skills and develop their moral virtues, helping them preserve a sense of purpose, and supporting the integration of caregiving with other aspects of caregivers’ personal lives. We begin by outlining the conditions under which work is meaningful, and we then apply this framework to caregiving. Subsequently, we evaluate how SARs influence these conditions, identifying both opportunities and risks. We end by briefly considering some practical implications pertaining to SARs design meant to foster meaningful caregiving practices.

Still, one could ask, why talk about human caregivers if SARs are built precisely to replace them? Our emphasis is still on human caregivers—both formal (paid workers) and informal (unpaid family or community members)—since robotic technologies continue to face significant challenges concerning autonomy, navigation, and mobility, which restrict their ability to completely substitute human caregivers. The integration of SARs into caregiving matters because it directly addresses the growing demand for caregiving services, particularly as life expectancy continues to rise. SARs offer the potential to improve caregiving outcomes by assisting in various capacities across formal, informal, and home-based care settings, as we show in what follows. The question, therefore, is not simply whether these technologies can be integrated, but how they can be designed and used to support the specific caregiving needs of older adults and those who care for them. More precisely, we claim that for the foreseeable future, human caregivers will remain integral to older adults’ care, working alongside SARs in both formal and informal settings. Understanding how such human–robot collaborations might affect care work is essential, particularly since how care work is perceived by workers profoundly shapes both caregivers’ sense of meaning and the overall quality of care that older adults receive.

These considerations are more and more timely in the wake of global attempts to design environments for healthier, more autonomous, and more dignified aging through the integration of smart technology. Referred to as smart, healthy, and age-friendly environments, these are attempts to bring together architectural design, digital innovation, and social support systems in order to enhance the quality of life for older adults (Dantas et al. 2019; Kavšek et al. 2021; Facchinetti et al. 2023; Sourbati and Behrendt 2021). Though a good deal of this research is directed at physical safety, mobility, and access to care, it is also important to ask how these environments impact those doing the caring. In this instance, if SARs are to become integral parts of age-friendly caring environments, their design has not only to address the needs of older adults themselves but also the moral and psychological well-being of carers. This paper draws attention to this relatively neglected aspect: how technological innovations in care can facilitate or obstruct the practice of caregiving as meaningful work.

## Work and meaningful work

### Defining work

What is work? This is a difficult question to answer, inasmuch as work involves many types of activities that require different skills and capacities. Some individuals work by themselves, while others have to coordinate with large teams. Certain forms of work involve cognitive labor, whereas others are largely physical. Work may produce tangible objects, such as physical goods, or intangible goods, such as artistic performances or specialized services. The definition of work also varies across cultures and historical periods (Grint 2005). Activities that seem work for some, such as childrearing or caregiving, are for others moral obligations that can be subtracted from the realm of work. Nor is work necessarily just about getting paid; one could not just say that someone who takes care of the house and raises kids is not working. Work goes beyond activities we do in exchange for money or for producing tangible things.

Despite these complexities, we can differentiate work from nonwork activities, such as leisure. This is because “work aligns with the concepts of production, contribution, and effort” (Veltman 2016, 24). This means that work, unlike leisure, addresses wants and needs, either individually or collectively, which presupposes some level of effort. More generally, work is a goal-oriented activity with the purpose of producing something of value. So, a very broad definition of work stipulates that work is any activity that is purposeful, productive, and goal-oriented and which requires sustained efforts and adds something of value to the social world.

Work is an activity that affects the person doing it beyond the extrinsic benefits it offers, such as wages, access to health care, or social status. What happens at work and the work we do affect us psychologically. The possibility to exercise independent judgment, develop skills and abilities, and see one’s added value can contribute to positively evaluating one’s life. Moreover, work can also offer a sense of purpose and personal fulfillment, as it can provide the conditions for self-expression and self-development, alongside a sense of usefulness for one’s community or world at large (Gheaus and Herzog 2016). In other words, when work is meaningful, it positively contributes to workers’ well-being. This raises the question: What makes work meaningful?

### Meaningful work

To begin with, the concept of ‘meaning’, whether in life or in work, is multidimensional, realized in multiple forms, and depends not on one, but on several factors that involve human values and purposiveness. Moreover, any attempt to answer the question of what makes work meaningful cannot escape the subjective/objective distinction. Like Veltman, we claim that meaningful work is one that feels good for the worker, thus is subjectively good, but also contributes something objectively valuable to the world. Objective here means independent of the beliefs and desires of the worker (Veltman 2016, 20). For example, raising a child, even if one sees it as tedious, is still objectively valuable, because “nurturing a child […] bears value even if a parent should unfortunately fail to see this is so” (Veltman 2016, 113). Thus, meaningful work has both objective and subjective dimensions, in that it takes into account both the phenomenal experience of the worker as well as the contributions of the work to the outside world or to the development of the worker.

There are four key conditions that confer meaning to work: skills, virtues, purpose, and integration of professional and personal life. But they do not exhaust the sources of purposefulness (Veltman 2016, 118-135), and which can be found in several accounts of and attempts to define meaningful work (Smids et al. 2020; Gheaus and Herzog 2016; Veltman 2016):

1. **Skills**: Work is meaningful when it offers the possibility to exercise skills, while also contributing to workers’ recognition by others and to their self-esteem. This is confirmed by psychological studies indicating people’s need to feel competent (Ryan and Deci 2002). But in order to exercise or develop skills and capabilities, workers also need autonomy. In Attfield’s words, “only where the activity reflects skill or judgment on the workers’ part can it embody the worker’s own standards” (Attfield 1984, 143). When the procedures and rhythms of work are exclusively determined by others, the worker can hardly identify with what they do, which can affect (self)esteem and recognition, on which “human beings have a constitutional dependence” (Veltman 2016, 121). When one can exercise independent judgment and develops their skills, this contributes to their self-esteem and can provide opportunities for garnering social recognition.
2. **Virtues**: Work that contributes to the development of virtues, principally of self-respect, honor, dignity, empathy, or reciprocity, is work that gives purpose to workers’ lives. For Veltman, “work appears a predominant venue for a range of virtues, including honor, dignity, pride, dependability, industriousness, cooperativeness, self-discipline, and self-reliance” (Veltman 2016, 120), although one can acquire virtues even outside work contexts. But given that work occupies most of our waking adult life, it becomes an important source for the development of virtues or of what Gheaus and Herzog (2016) call excellence. Excellence represents a regulative ideal that can manifest in various forms and sizes, encompassing achievements in knowledge, technology, esthetics, and more. In simple terms, work is considered objectively meaningful when it serves a greater good and facilitates personal growth, aiding individuals in becoming better at what they do and when what they do adds something of value to the world.
3. **Purpose**: Work that has a useful purpose and produces something of value for society, a community, or just for the worker, can be said to be meaningful (Veltman 2016, 136). Through work, people can feel they contribute something to the world, which makes life easier and more palatable, despite hardships and difficulties, as it can give one a sense of direction and purpose. Work can thus accomplish a purpose either for others or for the worker, which allows for meaningfulness in a wide range of occupations, including physical work or craftsmanship. For instance, the work of a mobster, which involves extortion or violence, is not meaningful, even if the mobster hones their skills and becomes highly proficient in illegal activities. So, meaningful work aligns with an ethical life, whereas work pursued for immoral ends cannot be meaningful.
4. **Integration of personal and professional life**: Work that is seemingly integrated and integrates the workers’ life, either by reflecting personal values and commitments or by embedding the worker in a relational context in which they identify as meaningful. Creating relationships with others or with places (such as people that work to protect the environment), feeling that one’s work adds value to the social world, and getting recognition for it, can be an important source of meaning and of well-being. But when work has to be hidden from others, it adds extra layers of burden in people’s lives. Take here the case of sexual workers, who most of the time hide their profession from their friends and families; in these situations, sexual work is accompanied by a whole host of social and political taboos, which leads to a schism between the professional and personal life of workers, in the end, negatively impacting workers’ well-being (Benoit et al. 2018).

Work can, of course, have multiple other sources of meaning, or meaning can be derived from just one factor amongst those claimed above. For example, one can have a sense of contributing something of value to the world, even though they do not have the opportunity to exercise their skills or develop virtues. And one can be proud of what one does, even if the work provides little opportunity for self-expression. It might also be the case that work is only extrinsically valuable, for example, when one works just to support a family. But work that is both intrinsically and extrinsically valuable, when it contributes something of value but also provides opportunities for the worker to flourish, is work that is *robustly* meaningful and that contributes to workers’ well-being.

Overall, meaningful work is both good for the worker and beyond, as it offers conditions for subjective fulfillment and objective contributions to the world. The four conditions for meaningful work offer a useful framework for testing which types of work are meaningful, contributing to workers’ well-being. As we will see in the next section, caregiving to an older person provides a compelling case for examining these dimensions, particularly because it is frequently overlooked or undervalued as a form of work.

## Meaning in caregiving

### Is caregiving work?

Caregiving comes in many shapes and sizes and requires different levels of specialization and skill. There are two types of care work for the benefit of older adults. Formal (paid) caregiving is performed in public or private hospitals, nursing homes, or care establishments as well as in private households. In these cases, care workers are in an official employment relationship and are unrelated to the persons they tend to (Addati 2018a, 7). Informal (unpaid) caregivers, on the other hand, provide care to family members or members of their community, on the basis of prior personal affective relationships. Needless to say, oftentimes it is women who take on caregiving for family members (Ophir and Polos 2022; Ehrlich et al. 2020).

Both paid and unpaid caregiving presuppose “activities and relations involved in meeting the physical, psychological, and emotional needs of adults and children, old and young, frail and able-bodied” (Addati 2018b, 4). Both activities^1^ require engaging with care recipients, most often in sustained care relationships. In order to care for older persons, with impaired mobility or declining cognitive capacities, the caregiver has to exercise a set of helping skills and specialized knowledge, such as symptom management, personal care delivery, or handling emotional distress. So, it seems that despite common conceptions, caregiving, both paid and unpaid, is work. It is an activity that is goal-oriented (assisting the care-receiver in daily life), purposeful (enhances the quality of life of the care-receiver), requires sustained efforts on the part of the caregiver (development of skills and emotion regulation) and adds value to the social world by providing a substantial contribution to countries’ economies, as well as enhancing individual and societal well-being.

### Meaning in caregiving

The issue of meaning in caregiving work is often overlooked in public discourse. This is because both paid and unpaid care work for the aging population lacks social recognition. Unpaid care work is often seen as a moral obligation rather than work, disproportionately burdening women (Addati et al 2018a). Additionally, there is a lack of compensation in terms of social security or health insurance for this type of work (Carmeli 2014). Informal care work often remains “invisible” and fails to receive recognition for its significant contribution to society (Fineman 2000). A report from the International Labor Organization from 2018 estimated that approximately 2 billion people work in informal care settings for approximately 8 h per day; valued on the basis of an hourly minimum wage, that amounts to US$11 trillion (Addati et al. 2018a, xxix). Similarly, paid caregiving, such as low-paying jobs in nursing homes or those provided by migrants as home care providers, also suffers from a lack of social recognition. In many societies, caregiving is undervalued, resulting in caregivers receiving less recognition and lower compensation compared to professions such as doctors or professors, which are typically seen as generating greater social value (Addati et al. 2018a).

While caregiving clearly meets the criteria for work, understanding the specific factors that make it meaningful requires a closer look at the daily realities and experiences of caregivers. In what follows, we rely on empirical studies (not exhaustive) to uncover under what conditions both formal and informal caregivers derive purpose, satisfaction, and a sense of fulfillment from their caregiving roles.

1. **Skills**: From empirical research, there is evidence that the development of personal knowledge, capabilities, and skills is an important source of meaning in caregiving (Pavlish et al. 2019; Vidman and Strömberg 2017; Al-Janabi, Coast, and Flynn 2008; Yates, Tennstedt, and Chang 1999). Both formal and informal care work vary from day to day and presuppose dealing with various challenges and unexpected situations. This variability requires identifying means or ways to overcome challenges, which requires a constant improvement of one’s skills and innovative applications of existing ones (Pavlish et al. 2019; Vidman and Strömberg 2017; Eldh et al. 2016). Another important source of meaning identified by care workers that allows them to independently exercise their knowledge and judgment is autonomy. Whether understood as professional decision-making (in the case of paid care workers) or freedom or control over the work environment (in the case of unpaid care workers), autonomy allows caregivers to express their full potential (Finn 2001).
2. **Virtues**: Caregiving, whether at home or in medical settings, fosters the development of moral virtues, such as empathy and reciprocity, and, implicitly, positively contributes to the development of moral character (Vallor 2017). Caregiving is, when done well, an activity that ‘trains’ the caregivers’ empathy, as when caring for older people, the disabled, or the sick, one has to recognize, feel, and respond appropriately to the suffering and pain of the other (Vallor 2017, 259). This is also confirmed by empirical research showing that when caregivers develop empathy, job satisfaction increases while burnout decreases (Yue et al. 2022). However, it is important to recognize that caregiving can also be emotionally draining and burdensome, especially when caregivers face high levels of stress, lack of support, or when the demands of caregiving become overwhelming. Under these conditions, caregiving can contribute to what some researchers refer to as ‘empathy fatigue’, where caregivers experience emotional exhaustion and a decrease in their ability to empathize with care recipients (Pomponi et al. 2016; Hua et al. 2021; Sim et al. 2023). Thus, while caregiving has the potential to develop empathy and other virtues, it can also lead to negative emotional outcomes when caregivers are not adequately supported.
  What is more, caregiving teaches that we have to be there for those who were there for us in times of need; but more than that, it creates the expectation that just as we tend for older people, so when we will be older someone will tend for us as well (Vallor 2017). Also, caregiving requires caregivers to show concern for other human beings and to treat them with respect (Voinea et al. 2022). In other words, caregiving can be meaningful as it provides caregivers the possibility to exercise virtues and develop their moral character.
3. **Purpose:** Empirical research indicates that meaningfulness in caregiving, both paid and unpaid, derives, to a great extent, from the development of caring relationships with care recipients, which foster recognition, value, and significance (Mishra et al. 2023). For instance, Pavlish et al. (2019) show that caregivers, both formal and informal, feel their work is meaningful when they have a sense of making important and positive contributions to a worthwhile purpose, such as building caring relationships and improving the lives of care recipients. Especially in care work, meaningfulness can be disconnected from happiness, as it is associated with benefiting others, while happiness relates to personal satisfaction (Pavlish et al. 2019). It is possible that in caregiving contexts, efforts to benefit care recipients can be stressful and resource-consuming, which may decrease happiness (Pavlish et al. 2019). Additionally, the well-being of care recipients may decline regardless of the care provided, which can also impact happiness (Baumeister et al. 2013). Nevertheless, even in these challenging situations, care work can still be meaningful. Similar conclusions were reached by Vidman and Strömberg (2017) in their study on meaning in care work. They found that paid care workers perceive their work as meaningful when they are able to enhance the lives of care recipients. There is a feedback loop, inasmuch as when caregivers “think they can make life better for older people, the staff feel good and if the staff are satisfied, older people are satisfied as well” (Vidman and Strömberg 2017, 113).
4. **Integration of personal and professional life**: Carers, both formal and informal, do not feel valued, seen, or heard within their communities, and many report a lack of recognition of their expertise (Seddon and Robinson 2015; Hamilton et al. 2024). Unpaid care work is often viewed as a moral obligation disproportionately burdening women. Additionally, there is no compensation in terms of social security or health insurance (Carmeli 2014). Informal care work often remains “invisible” and fails to receive recognition for its significant contribution to society (Fineman 2000). Similarly, paid caregiving is undervalued, so caregivers receive less recognition and lower compensation compared to professions such as engineers or teachers, which are typically seen as generating greater social value. In other words, because caregiving is not socially recognized and is even stigmatized, it tends to be less appealing to people and less easily integrated into one’s life (McAllum et al. 2024).

Although it has the potential for being fulfilling, caregiving is beset by many challenges. There appears to be a direct link between suboptimal working conditions, caregivers’ low job satisfaction, and a decrease in the quality of the care being delivered. Dissatisfied caregivers are likely to exhibit signs of emotional exhaustion, burnout, and diminished commitment to caregiving duties. Consequently, the quality of care delivered could decrease, affecting the health, well-being, and outcomes of care recipients. On the other hand, caregivers with higher job satisfaction tend to show better emotional states, empathy, and commitment towards their duties (Ejaz et al. 2008). The problem of finding meaning in caregiving is thus not merely of concern to caregivers themselves, but also to care-receivers who are inevitably affected by the performance of those responsible for their care.

## Social robots, caregiving, and meaningful work

SARs hold the potential to ease demanding or repetitive tasks, possibly freeing caregivers to focus more on the fulfilling aspects of care, while also easing the stress that often comes with caregiving. However, as we will show, the adoption of SARs also transforms how caregivers do their work, engage with older adults, and experience the meaningfulness of their role. In what follows, we turn our attention to whether and how SARs might enhance or undermine the dimensions that make caregiving a fulfilling and morally significant activity (see Summary Table 1).

**Table 1:** Meaningful work and SARs.

Conditions for meaningful work	Positive effects of SARs	Negative effects of SARs
Skills	- Reduce repetitive tasks - Enhance technical expertise and professional recognition	- Increased workload or alter nature of tasks - Caregiver may feel undervalued if SARs take over relational tasks - SARs may require additional caregiver training and supervision
Virtues	- More time for empathy, patience, and reciprocity - Ability to reflect on ethical responsibilities	- Reduced face-to-face interaction may hinder empathy - Risk of emotional distance from care recipient
Purpose	- Ability to meet care recipients’ needs more effectively - Enhanced caregiver satisfaction by providing time for respite	- Loss of caregiver purpose if SARs displace meaningful tasks - May reduce the caregiver’s sense of contribution
Integration of personal and professional life	- Reduced physical strain, enabling better work-life balance. - More time for personal commitments	- Technical maintenance could increase caregiving stress - Additional stress, disrupting the harmony between personal and professional caregiving roles.

### Skills

#### Positive

Two scoping reviews investigating the influence of robots on the work environment of paid caregivers revealed that robots can lead to a reduction in the amount of physical workload (Persson et al. 2022; Papadopoulos et al. 2018). By offloading tasks such as lifting or closely supervising older adults, SARs may free caregivers to develop and practice skills they consider most meaningful, such as offering emotional or psychological support to care recipients (Louie and Nejat 2020; Jung et al. 2017). What is more, operating SARs can offer caregivers the opportunity to develop technical skills, which could contribute to enhancing their professional recognition.

#### Negative

On the other hand, robots might not necessarily reduce workload but instead alter the nature of work or potentially increase the workload of care workers (Persson et al. 2022). For instance, studies examining robotic arms that assist older adults in eating (Dage et al. 2017), personal hygiene (Beedholm et al. 2015), or mobility (Kapsalyamov et al. 2019) showed that although robots can enhance efficiency in assisting with purely physical tasks, caregivers still need to supervise and assist older adults in using these technologies. Caring for and troubleshooting SARs could potentially impose an additional responsibility on caregivers, who might also confront obstacles in terms of lacking requisite technical support or access to the necessary resources for acquiring proficiency in operating and solving issues related to SARs (Gul et al. 2024; Pino et al. 2015). Also, caregivers may feel their contributions are undervalued if SARs take over visible aspects of caregiving (Papadopoulos et al. 2018).

### Virtues

#### Positive

By reducing physically strenuous or repetitive tasks, SARs may free caregivers to invest themselves more fully in the relational aspects of care, potentially cultivating virtues like empathy, patience, and reciprocity (Persson et al. 2022; Greenhalgh et al. 2019). Also, by taking on some tasks that are burdensome, such as lifting, entertaining, or answering repetitive questions, caregivers may find time to attend to the emotional well-being of care recipients and their own need for occasional respite (Persson et al. 2022). This time off could potentially foster deeper, more empathic interactions and could help caregivers better reflect on their responsibilities.

#### Negative

However, delegating tasks to robots might increase the distance between caregivers and care recipients, as the possibilities for physical touch and personal time with older individuals diminish (Wright 2023). Interestingly, Pigini et al. (2012) show that informal caregivers do not appreciate the possibility of SARs having direct physical contact with care recipients. This perspective aligns with concerns expressed by paid caregivers, who also worry that the introduction of new technologies might diminish the human aspect of caregiving, potentially making their work feel less meaningful by taking away the opportunities to develop and practice moral virtues (Sriram et al. 2019).

### Purpose

#### Positive

SARs can also enhance caregivers’ sense of purpose by helping them meet older adults’ needs more effectively. Many informal caregivers see value in offloading routine tasks—such as fetching items, monitoring vital signs, or entertainment—to free them for more meaningful interactions (Pigini et al. 2012). In this way, SARs could preserve or even enhance caregivers’ sense of purpose, provided they are used as tools to support, rather than replace, the meaningful relationship-building aspects of care work.

#### Negative

However, research shows that SARs sometimes add to caregivers’ responsibilities instead of reducing them. Wright concludes, after an extensive ethnographical study examining the introduction of SAR in Japanese care homes, that these technologies require extra-work on the part of caregivers, finally taking a toll on them, by cutting their time for building caring relationships with older individuals: “Instead of replacement, a better description for the relationship between care robots and human caregivers is displacement. The introduction of care robots displaces skills, practices, and value; their mediation displaces direct human-human contact; caring for robots risks displacing caring for people” (2023, 132). Wright observed that while service robots, such as Hug, reduce the need for human touch and closeness, social robots, such as Paro, reduce the time for caregivers to interact one-on-one with care recipients. If caregivers, both paid and unpaid, become managers of SARs, spending less and less time with older individuals, thus having less possibilities to build caring relationships, then they might lose track of what really matters in their work: “more generally, if robots reduce workers’ contributions to worthy causes, they may lose purpose.” (Smids et al. 2020, 522). This shows that both paid and unpaid caregivers need to see the added value of their work in the life of the care recipient, and SARs are accepted only inasmuch as they have the potential to enhance this aspect.

### Integration of personal and professional life

#### Positive

There is evidence that assistive technologies, such as SARs, can assist unpaid caregivers in reclaiming time for themselves, alleviating stress, as well as gaining a crucially needed break in the form of respite (Dembovski et al. 2022; Sriram et al. 2019; Jenkins and Draper 2015). Caregivers may more readily achieve a balance between family, social, and recreation commitments, on the one hand, and caregiving, on the other, if robots can take on repetitive or labor-intensive tasks. This break, in its turn, could promote a better work-life balance as well as allow caregivers to continue in their caregiving role in the long term. Without respite, caregivers can become burned out, decreasing intrinsic rewards, as well as impacting the caregiving experience negatively.

#### Negative

Although SARs can minimize some manual labor, the necessity of dealing with technical issues and constantly monitoring SARs' interactions can erode caregivers’ emotional spacing necessary for reflection or recuperation from the demands of work (Wright 2023). Thus, while SARs bring valuable dividends in terms of alleviating caregiving activities, their design and interface are key determinants of their usefulness. The ease or difficulty of a SAR’s user interface can either assist or hinder the caregiver. If the interface is overly complex or not intuitive, caregivers might experience additional stress in learning how to use the technology, which would take away from the very respite that SARs could potentially deliver. Such additional intricacy could add a burden for caregivers. This dynamic could blur the divide between professional and personal life, aggravating burnout if caregivers are kept constantly on-call in order to address technical failures.

Despite the potential benefits of SARs for both paid and unpaid caregivers, we also see some potential worries. If caregivers become mere ‘machine operators’, then this might affect the well-being of caregivers and, implicitly, the well-being of care recipients, as care work might become “increasingly deskilled, devalued and alienated” (Wright 2023, 70). On the other hand, if these robots are designed so as to draw people more into caregiving practices, by offering limited support for repetitive, difficult tasks, then these technologies could enhance the possibilities for finding meaning in caregiving.

## Conclusion and design implications

This paper adds a contribution to the debates about how to create smart, healthy, age-friendly environments by foregrounding the experiences and moral needs of human caregivers. We argued that SARs should be designed not only to assist older adults with daily tasks, but also to support the conditions under which caregiving becomes meaningful—through skill development, the development of moral virtues, a sense of purpose, and better integration of personal and professional life. While smart, healthy, age-friendly environments initiatives often focus on how technologies can optimize living conditions for older adults, we suggest that this cannot be achieved sustainably without attending to the moral and emotional ecology of care work itself.

We are still quite far from the futuristic scenarios where robots can be completely autonomous and can be trusted with the care of people unsupervised. As showed above, robots need to be manipulated by humans which means that they might inadvertently add new tasks such as “setting up, configuring, moving, operating, mediating, storing, cleaning, maintaining, updating, managing, and overseeing them—in other words, taking care of them” (Wright 2023, 141). If caregivers end up spending more time on caring for SARs, then they have less time to care for older adults. This, as we argued above, might make their work less meaningful, which will affect not only the caregivers’ well-being, but also that of care recipients.

This is because there is a distinction between care and *caring*, and human beings are more than just “objects” that have to be fed, cleaned, moved around, and entertained. Older adults, just like all people, have to be treated with respect, which entails “taking their interests into account, helping them when possible, or acting with their well-being in view” (Voinea et al. 2022). For the moment, we know that robots cannot accomplish this, which does not mean the fact that in the future they will not develop these human-like empathic and moral capacities. But it doesn’t make sense to ignore today’s reality, where robots are working in collaboration with humans, in order to prepare for a future that might or might not actualize. For these reasons, we should pay more attention to how SARs are designed and how they will change the caregiving landscape. Given that when caregiving is meaningful, it positively impacts the well-being of both caregivers and care recipients, SARs should be designed specifically for fostering the conditions conducive to meaningful care work.

How are SARs designed, and whose interests and what purposes do developers have in mind? The literature shows that there is often a disconnect between developers and the actual beneficiaries of these technologies, meaning older adults and their caregivers. More precisely, developers sometimes, while aiming to solve specific care-related challenges, do so without adequately considering the real preferences, desires, and experiences of those they are designing for: “most care robots were never developed specifically with care work in mind—the solution came before a consideration of the problem and was adapted to fit retrospectively.” (Wright 2023, 19). Already, researchers such as Tanioka et al. (2021a; 2021b), Betriana et al. (2021), and Pepito et al. (2020) to name just a few, have advanced ways of designing SARs that directly address the needs of older adults, such as enhancing compassionate communication and developing empathetic, affective robots; but, as Wright (2023) documents in his ethnographic study, these suggestions are still not always adequately integrated into design process.

The development of socially assistive robots (SARs) for older adults can lead to the design of paternalistic technologies, underpinned by a belief that older individuals are inherently incapable of managing their care or making decisions independently (Voinea, Wangmo, and Vică 2024; Neven and Peine 2017). SARs and other technology used in caregiving contexts are frequently based on negative attitudes toward aging, in which older adults are viewed as frail, passive, and in need of dependence upon others for health (Neven and Peine 2017; Neven 2015). This can lead developers to design robots centered on the physical needs of older adults, such as mobility or health monitoring, rather than the more complex, relational, and emotional components of care necessary for maintaining independence, dignity, and control in aging (Rubeis 2020). Consequently, robots can inadvertently perpetuate stereotypes, reducing the opportunity for older adults to exercise control or have a sense of engagement in their care (Chen 2020; Rubeis 2020; Voinea et al. 2024).

So, how should SARs be developed to foster meaningful work? Below, we build upon recommendations from existing literature and also propose additional directions for future development:

- **Empower rather than displace caregivers**: SARs should be designed to transfer labor-intensive or physically challenging tasks (e.g., lifting or surveillance) in order to free up caregivers to devote themselves to empathetic, relational, and judgment-based activities.
- **Foster skill development**: By providing user-friendly interfaces and flexible features, SARs can assist caregivers in honing and broadening their professional skills instead of completely delegating them to machines.
- **Preserve human connection**: SARs should support, not substitute, face-to-face interaction and social support, allowing caregivers to continue having close, personalized relationships with older adults. This is sustained by the research of Tanioka et al. (2021a, 2021b), who propose that future social robots should be equipped with more effective communication functions with the capability of better dealing with human emotional needs.
- **Enhance sense of purpose**: The developers should aim at legitimate care needs (e.g., fall prevention or social isolation reduction) and identify specifically how SARs might assist caregivers in ensuring the better well-being of care recipients. For instance, fall prevention by assistive technology has been addressed by Miyagawa et al. (2019), who also underscore the necessity of addressing such practical, safety-related issues, aligning with our suggestion regarding SARs addressing immediate care needs.
- **Facilitate work–life balance**: While reducing physical strain, SARs must avoid introducing excessive technical upkeep or monitoring burdens; easy-to-use systems and ready support help caregivers integrate work and personal life seamlessly.

In this paper, we argued that when care work is meaningful, it positively impacts the well-being of both care providers and care recipients. Consequently, we have moral reasons to build SARs that facilitate the cultivation of meaningful work, as this has the potential to enhance the quality of life for both caregivers and care recipients. This is a novel proposition, inasmuch as SARs are normally designed to solve or manage very specific tasks, without too much consideration from the part of developers to how people who will actually work side-by-side with these technologies are affected by them. We need more proactive thinking about how to build a world where technology serves people, and not the other way around. This paper is meant to be a step in this direction.

## Data Availability

No datasets were generated or analyzed during the current study.

## Footnotes

1 There are also some differences between the two – for example, paid caregiving means that the caregiver should tend to more individuals and thus, has less time for developing meaningful relationship with each care recipient. But paid caregivers also have the possibility to take time off. For unpaid caregivers, oftentimes tending for a family member, there is more time to learn what the care-recipient needs and to provide it, although there is almost no time off. See Dixe, M. dos A.C.R., da Conceição Teixeira, L.F., Areosa, T.J.T.C.C., Frontini, R.C., de Jesus Almeida Peralta, T., & Querido, A.I.F. (2019). Needs and skills of informal caregivers to care for a dependent person: a cross-sectional study. *BMC Geriatrics*. *19*(1), 255. 10.1186/s12877-019-1274-0.
